# Different settings of place of midwife-led birth: evaluation of a midwife-led birth centre

**DOI:** 10.1186/s40064-016-2306-2

**Published:** 2016-06-18

**Authors:** Jacoba van der Kooy, Johanna P. de Graaf, Doctor Erwin Birnie, Semiha Denktas, Eric A. P. Steegers, Gouke. J. Bonsel

**Affiliations:** Division of Obstetrics and Prenatal Medicine, Room Hs-408, Department of Obstetrics and Gynaecology, Erasmus MC, PO Box 2040, 3000 CA Rotterdam, The Netherlands; Institute of Health Policy and Management, Erasmus University Rotterdam, PO Box 1738, 3000 DR Rotterdam, The Netherlands; Rotterdam Midwifery Academic (Verloskunde Academie Rotterdam), Dr. Molewaterplein 40, 3015 GD Rotterdam, The Netherlands; Department of Public Health, Erasmus MC, PO Box 2040, 3000 CA Rotterdam, The Netherlands

**Keywords:** Birth centre, Maternal outcome, Midwife-led care, Perinatal outcome

## Abstract

**Objectives:**

The claimed advantages of home deliveries, including fewer medical interventions, are potentially counter balanced by the small additional risk on perinatal adverse outcome compared to hospital deliveries in low risk women. Homelike birth centres have been proposed a new setting for low risk women combining the advantages of home and hospital, resulting in lower intervention rates with equal safety. This paper addresses whether the introduction of a midwife-led birth centre adjacent to the hospital combines the advantages of home and hospital deliveries. Additionally, we investigate whether the introduction of a midwife-led birth centre leads to a different risk selection of women planning their delivery either at home, at the hospital or at the birth centre.

**Methods:**

Anonymized data, between January 2007 and June 2012, was collected from the four participating midwife practices. Women (n = 5558) were categorized according to intended place of birth. Women’s characteristics and pregnancy outcomes were compared between the period before and after its introduction using Chi square and Fisher’s Exact tests. Direct and indirect standardized rates were calculated for different outcomes [(1) intrapartum and neonatal mortality (<24 h), (2) composite outcome of neonatal morbidities, (3) composite outcome of maternal morbidities, and (4) medical intervention], taking the period before introduction as reference.

**Results:**

After the introduction of the birth centre a different risk selection was observed. Women’s characteristics were most unfavourable for intended birth centre births. Additionally, an higher neonatal risk load was seen within these women. After its introduction neonatal morbidities decreased (5.0 vs. 3.8 %) and maternal morbidities decreased (8.3 vs. 7.3 %). Interventions were about equal. Direct and indirect standardization provided similar results.

**Conclusion:**

Neonatal morbidity and maternal morbidity tended to decrease, while overall intervention rates were unaffected. The introduction of the midwife-led birth centre seems to benefit the outcome of midwife-led deliveries. We interpret this change by the redistribution of the higher risk women among the low risk population intending birth at the birth centre instead of home.

## Background

There are considerable variations in organization of Perinatal Care. In the Netherlands, approximately 50 % of women start delivery under supervision of a community midwife. Dutch community midwives are independent health care professionals who provide care for low risk and medium risk pregnant women. Dutch guidelines define low, medium and high risk pregnant women (CVZ. CV 2003). A low risk pregnant woman who becomes high risk is referred antenatally or during delivery to the gynaecologist for remaining ante- and intrapartum care. Low risk women are allowed to choose the place where to deliver: at home, in the hospital or in a birth centre, all supervised by the community midwife. The frequency of these different midwife-led birth places differs across regions, with fewer home deliveries in urban areas (Nederland SPR 2011). Pregnant women with so called medium risks should deliver in the hospital according to Dutch guidelines, yet supervised by the midwife only.

One of the concerns raised regarding the Dutch Perinatal Care System is whether the small additional risk on adverse perinatal outcome (perinatal mortality and intrapartum related neonatal morbidities) of home deliveries is of fact by the claimed advantage of lower intervention rates compared to planned hospital births (Ackermann-Liebrich et al. 1996; Hutton et al. 2009; Janssen et al. 2002; Lindgren et al. 2008; Olsen 1997; Wiegers et al. 1996; van der Kooy et al. 2011). Birth centres adjacent to hospitals have been proposed a new setting that combines the advantages of both home and hospital, resulting lower intervention rates with equal safety (Chervenak et al. 2013; Hodnett et al. 2005; Hundley et al. 1994; Waldenstrom et al. 1997). They are designed to provide an intermediate option of care between home and hospital birth for low risk women. Despite existing organization differences, birth centre care generally includes a homelike, nonclinical environment, a rather autonomous midwifery practice, and a commitment to and belief in normal, physiologic birth (Hodnett et al. 2005; Hundley et al. 1994; Coyle et al. 2001).

In this paper we address whether the introduction of a midwife-led birth centre adjacent to the hospital combines the advantages of home and hospital deliveries. This was done by comparing the regional perinatal outcomes before and after the introduction of the birth centre hereby comparing the different places of birth. Additionally, we investigate whether the introduction of a midwife-led birth centre leads to a different risk selection of women planning their delivery either at home, at the hospital or at the birth centre, resulting in an altered risk for perinatal, maternal morbidities and intervention pattern.

## Methods

### Birth centre

The birth centre Sophia started care in October 2009. It is a separate unit, consisting of four single birthing rooms and 12 rooms for post partum maternity care. It is located on the same floor as the obstetric labour ward (100 m), yet with its own entrance and home-like interior. The unit is staffed by local community midwives. The unit fits to the UK National Perinatal Epidemiology Unit report description of a ‘midwife-led birth centre adjacent to the hospital’, i.e. “An alongside midwifery care unit offering care to women with straightforward pregnancies during labour and birth in which midwives take primary professional responsibility for care. During labour and birth diagnostic and treatment medical services, including obstetric, neonatal and anaesthetic care are available, should they be needed, in the same building, or in a separate building on the same site. Transfer will normally be by trolley, bed or wheelchair” (Coyle et al. 2001).

The aim of the unit is to provide a safe, homelike environment where low risk women can give birth. The unit aims to provide risk led care, including assessing the risk status of each women at different time intervals and act promptly and accordingly using standardised protocols. Special attention is given to certain ethnic minority groups and women with a low social economic background both in terms of risk monitoring and tailored provision of care. An expert group consisting of a gynaecologist, midwives, maternity nurses, a public health expert and administrating staff, is responsible for the continuous development, revision and extension of protocols and new interventions

Local community midwives take full responsibility for the care delivered, thus developing and maintaining their competence. Labour is managed traditionally; the fetal heart rate is monitored with a hand held Doppler apparatus, and interventions are minimal. When (acute) complications arise, obstetrical and neonatal expertise and clinical facilities are directly available.

### Design and data

A regional study in the north of Rotterdam was designed comparing outcomes before and after the introduction of the midwife-led birth centre in October 2009, using direct and indirect standardization. The four largest local community midwife practices in the region were approached, whose women are allowed to choose place where to deliver (in the birth centre, at home or in nearby hospitals). Anonymized data were collected from each of these registries. If women deliver in the birth centre, the data registration is still the midwife’s responsibility through internet entry. Data were collected between January 2007 and June 2012. We selected the records of all singleton pregnancies supervised by a community midwife at the onset of labour (5953 women). The onset of labour was defined as spontaneous contractions or the spontaneous rupture of membranes. Excluded were 62 women with so called ‘medium risks’ [e.g. women with a history of postpartum haemorrhage or obesity (BMI > 35)] and another 333 women since their planned place of birth was unknown. The remaining 5558 were divided into two groups, the period before the introduction of the birth centre (n = 1834) and the period after its introduction (n = 3724).

Within each period women were categorized by intended place of birth (at home, at the hospital or at the birth centre), which usually is concordant with the true place of birth.

The retrospective use of anonymized medical records exempted institutional review of the Medical Ethics Committee.

### Outcomes

Four primary outcomes were chosen: (1) intrapartum and early neonatal mortality (up to 24 h), (2) a composite outcome of intrapartum related neonatal morbidities (neonatal encephalopathy, brachial plexus injury, fractured clavicle, cephaloheamatoma, neonatal infection, low Apgar score (<7 after 5 min), neonatal hospital admission, or other trauma related to birth), (3) a composite outcome of intrapartum related maternal morbidities (third/fourth degree rupture or postpartum haemorrhage >1000 cc), and (4) the presence of a medical intervention (vacuum extraction, forceps extraction, or caesarean section).

### Data handling

Of the 27 selected variables, the variable education level had ≥30 % missing values and was therefore excluded from analysis. For all other variables missing values were <30 % and were replaced by mean, median and mode, for respectively, numeric (normally distributed), ordinal and nominal values.

### Data analysis

First, we compared women’s characteristics between the period before and after the introduction of the midwife-led birth centre, using Chi square tests and Fisher’s Exact tests. We compared women’s profiles before and after the introduction of the birth centre, according to the intended place of birth (see Table 1, column A; home before vs. after the introduction of the birth centre, and B; hospital before vs. after the introduction the introduction of the birth centre). Secondly, we compared women’s profiles for the different places of birth, after the introduction of the Sophia birth centre (see Table 1, column C; home vs. birth centre and D; hospital vs. birth centre).

**Table 1:** Characteristics of women in primary care at the onset of labour subdivided for planned place of birth and the period before and after the introduction of the midwife-led birth centre

	Before introduction of the birth centre				After introduction of the birth centre						Birth centre (N)	%	C	D
	Home (n)	%	Hospital (n)	%	Home (n)	%	A	Hospital (n)	%	B				
	443	21	1391	67	470	12		1583	41		1671	44		
Parity							*			*			*	*
Primiparous	246	56	850	61	191	41		852	54		970	58		
Multiparous	196	44	541	39	278	59		730	46		695	42		
Maternal age										*			*	*
<19 years	9	2	48	3	4	1		48	3		55	3		
20–25 years	28	6	185	13	24	5		142	9		205	12		
25–34 years	279	63	870	63	306	65		1028	65		1055	63		
>35 years	105	24	215	15	133	28		348	22		334	20		
Ethnic background													*	*
Western	351	79	723	52	352	75		783	49		584	35		
Non Western	50	11	416	30	64	14		419	26		869	52		
Neighbourhood													*	*
Privileged neighbourhood	293	66	802	58	321	68		963	61		790	47		
Underprivileged neighbourhood	150	34	589	42	149	32		620	39		881	53		
Married status										*			*	*
Married/living together	398	90	1173	84	435	93		1363	86		1303	78		
Single	37	8	218	16	30	6		180	11		340	20		
Education													*	
Low	na		na		7	1		20	1		52	3		
Middle					17	4		77	5		276	17		
High					73	16		138	9		337	20		
BMI														*
<20	67	15	205	15	79	17		230	15		251	15		
20–30	339	77	1048	75	350	74		1165	74		1281	77		
>30	26	6	120	9	23	5		133	8		100	6		
Folic acid use							*			*			*	*
No	76	17	338	24	46	10		264	17		303	18		
Preconceptional	53	12	177	13	126	27		349	22		429	26		
Only during pregnancy	297	67	823	59	283	60		939	59		896	54		
Antenatal care >14 weeks										*			*	*
No	359	81	1084	78	411	87		1353	85		1357	81		
Yes	84	19	307	22	59	13		230	15		314	19		
Gestational age										*				
<34 weeks	5	1	10	1	2	0		17	1		12	1		
35–36 weeks	4	1	19	1	4	1		32	2		33	2		
37 weeks	11	2	61	4	13	3		67	4		69	4		
38–41 weeks	422	95	1286	92	451	96		1464	92		1553	93		
>41 weeks	1	0	12	1	0	0		2	0		4	0		
Big3 outcomes														
SGA	29	7	107	8	23	5		121	8		120	7		
Premature	6	1	28	2	6	1		46	3		44	3		
Congenital abnomality	8	2	59	4	2	0		7	0		24	1		
Combination Big3	3	1	13	1	1	0		3	0		8	0		
Total Big3	46	10	207	15	32	7		177	11	*	196	12	*	

*na* not available

A = home before versus after the introduction of the midwife-led birth centre

B = hospital before versus after the introduction of the midwife-led birth centre

C = birth centre versus home (after the introduction of the midwife-led birth centre)

D = birth centre versus hospital (after the introduction of the midwife-led birth centre)

* p < 0.05 (Chi square test or Fisher exact test)

We calculated the detailed birth weight distribution [according to the national birth weight reference curves (Visser et al. 2009)] for all low risk singleton pregnant women, under supervision of a community midwife at the onset of labour between 2000–2007 in the municipality of Rotterdam (29,357 women), according to the planned place of birth. This served as reference for the birth weight distribution in the four practices, which we calculated for the periods before (1834 women) and after (3724 women) the introduction of the birth centre, again according to their intended place of birth.

Finally, we provided the prevalence of Big3 pregnancies as a proxy for risk load, which can be used to adjust for case mix differences. Big3 pregnancies are defined as: congenital abnormalities (list defined), intrauterine growth restriction (SGA, birth weight below the 10th percentile for gestational age, gender and parity specific), or preterm birth (<37th week of gestation) (van der Kooy et al. 2011). Detailed analysis of the complete perinatal dataset of the Netherlands Perinatal Registry (PRN), covering all pregnancies of the years 2000–2007 (1.25 million records), show that the presence of any of these three conditions preceded perinatal mortality in 80 % of cases (Bonsel et al. 2010). A p-value (two sided) <0.05 was considered a statistically significant difference.

The data were analysed with Statistical Package of Social Sciences version 20.0 for Windows (SPSS Inc, Chicago, IL, USA).

### Standardization

To increase the validity of our results of the comparison before and after the introduction of the birth centre, we applied direct as well as indirect standardization. The index population (i.e. the population of interest) consisted of the women who could plan their birth the midwife-led birth centre (n = 3724 of which 470 home, 1583 hospital and 1671 birth centre). The standard or reference population consisted of 1834 eligible women who could not plan their birth in the birth centre (443 planned home births, and 1391 planned hospital births).

In our analysis the index population was standardized according to the strata of the standard population. The index populations of planned birth centre births and planned hospital births were standardized using the standard populations of planned hospital births, since women’s profiles were similar. The index population of planned home birth was standardized using the standard population of planned home births.

Populations were stratified for parity (nulliparous vs multiparous), age (≤24, 25–34, ≥35 years), ethnicity (Dutch vs non-Dutch) and the presence of Small for Gestation Age (SGA, birth weight below the 10th percentile for gestational age, gender and parity specific; yes vs no). In our analysis the presence of SGA represented an objective estimate of the risk challenge at birth (van der Kooy et al. 2011; Bonsel et al. 2010).

The direct standardized rates were estimated as a weighted average of the index strata-specific outcome rates where the weights represent the strata-specific sizes of the standard population. The indirect standardized rates were estimated as the strata-specific outcome rates from the standard population to derive expected outcome rates in the index population for the four different outcomes. For both rates 95 % confidence intervals were calculated (Armitage and Berry 1994; Dobson et al. 1991; Kahn 1989).

We only presented direct standardized rates, unless indirect rates showed contrasting results.

## Results

Before the introduction of the Sophia birth centre, 443 (21 %) of women planned a home delivery and 1391 (67 %) planned a hospital delivery. After the introduction of the birth centre, 470 (12 %) women planned a delivery at home, 1583 (41 %) at the hospital, and 1671 (44 %) at the birth centre.

In the period after the introduction of the birth centre, women who planned birth at home were significantly more likely to be multiparous or had taken more often preconceptional folic acid (all favourable characteristics) compared to the women who planned birth at home in the period before the introduction (see column A). A similar pattern was seen in the women who planned their birth at the hospital. In the period after the birth centre introduction, these women showed more favourable characteristics compared to women in the period before the introduction. This group was significantly more often multiparous, older, married or living together, had taken preconceptional folic acid, and had received antenatal care before 14 weeks of gestation (see column B).

In the period after the introduction of the birth centre is was observed that women who planned birth at the birth centre showed more unfavourable characteristics compared to home as well as hospital birth [more likely to be nulliparous, of younger age, of non Western origin, from unprivileged neighbourhoods, single, did more often not take preconceptional folic acid, and more often received antenatal care after 14 weeks of gestation (see column C + D)].

An higher neonatal risk load was seen in women with a planned birth at the birth centre; the total prevalence of Big3 was higher (planned home 7 % vs. planned hospital 11 % vs. planned birth centre 12 %).

After the introduction of the birth centre, the intrapartum and early neonatal mortality decreased combining all deliveries regardless place (4/1834 = 2.2 ‰ vs. 2/3724 = 0.5 ‰).

Intrapartum related neonatal morbidities were significantly more common in the period before the introduction of the birth centre (91/1834 = 5.0 % vs. 140/3724 = 3.8 %; data not shown), as described in Table 2. After the introduction significantly more intrapartum related neonatal morbidities occurred in planned birth centre births compared to planned home or planned hospital births (5.3 vs. 1.9 vs. 2.7 % respectively).

**Table 2:** Outcomes of women in primary care at the onset of labour subdivided for planned place of birth and the period before and after the introduction of the midwife-led birth centre

	Before introduction of the birth centre				After introduction of the birth centre						Birth centre (N)	%	C	D
	Home (n)	%	Hospital (n)	%	Home (n)	%	A	Hospital (n)	%	B				
	443	21	1391	67	470	12		1583	41		1671	44		
Intrapartum related neonatal morbidies										*			*	*
Neonatal encephalopathy	0	0.00	0	0.00	0	0.00		0	0.00		1	0.06		
Brachius plexus injury	0	0.00	0	0.00	0	0.00		1	0.06		0	0.00		
Fractured clavicle	0	0.00	4	0.29	0	0.00		2	0.13		4	0.24		
Cephalohaematoma	0	0.00	0	0.00	0	0.00		0	0.00		0	0.00		
Neonatal infection	3	0.68	17	1.22	3	0.64		9	0.57		30	1.80		
Low Apgar score (<7 after 5 min)	4	0.90	24	1.73	4	0.85		20	1.26		29	1.74		
Neonatal unit admission	10	2.26	48	3.45	6	1.28		17	1.07		35	2.09		
Other trauma related to birth	0	0.00	4	0.29	0	0.00		0	0.00		3	0.18		
Total intrapartum related neonatal morbidities	13	2.93	78	5.61	9	1.91		43	2.72		88	5.27		
Intra partum and early neonatal death (24 h)														
No	441	100	1389	100	470	100		1582	100		1670	100		
Yes	2	0.45	2	0.14	0	0.00		1	0.06		1	0.06		
Intrapartum related maternal morbidies							*						*	
Postpartum haemorrhage >1000 cc	24	5	80	6	15	3		86	5		82	5		
Third/fourth degree rupture	16	4	33	2	9	2		35	2		43	3		
Total intrapartum related maternal morbidities	37	8	107	8	22	5		118	7		123	7		
Interventions										*			*	*
None	395	89	1166	84	420	89		1368	86		1403	84		
Vacuum or forceps extraction	34	8	159	11	35	7		134	8		173	10		
Caesarean section	14	3	66	5	15	3		81	5		95	6		

A = home before versus after the introduction of the midwife-led birth centre

B = hospital before versus after the introduction of the midwife-led birth centre

C = birth centre versus home (after the introduction of the midwife-led birth centre)

D = birth centre versus hospital (after the introduction of the midwife-led birth centre)

* p < 0.05 (Chi square test or Fisher exact test)

Intrapartum related maternal morbidities were also lower in the period after the introduction (153/1834 = 8.3 % vs. 270/3724 = 7.3 %). After the introduction of the birth centre they were highest among planned hospital births. Interventions were about equal in both periods (14.9 vs. 14.3 %). In the period after the introduction, interventions occurred significantly more often in birth centre deliveries compared to planned home or planned hospital births (16 vs. 11 vs. 14 % respectively)..

**Fig. 1:**
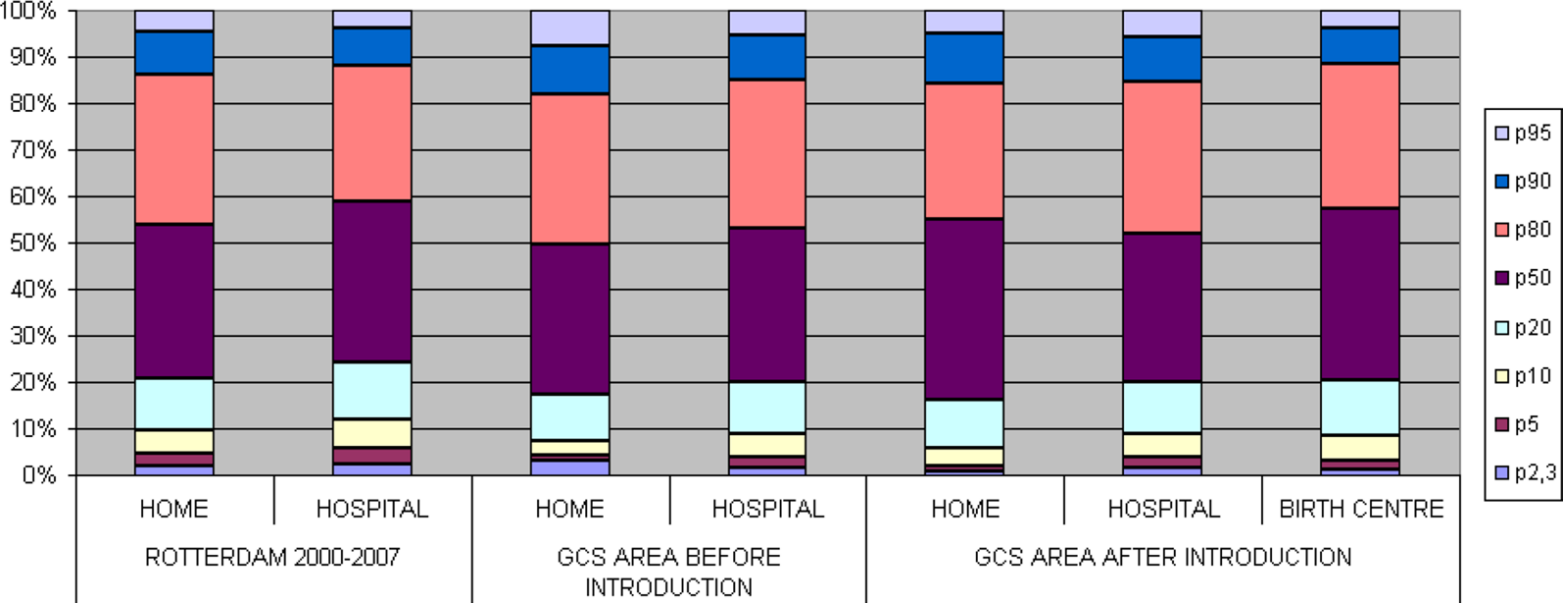
Birth weight distribution for all low risk singleton pregnant women, under supervision of a community midwife at the onset of labour, between 2000–2007 in Rotterdam, according to the planned place of birth

Figure 1 shows the birth weight distribution for all low risk singleton pregnant women, under supervision of a community midwife at the onset of labour, between 2000-2007 in Rotterdam, according to the planned place of birth. This was also shown for the periods before and after the introduction of the birth centre. In all periods, weights below the 10th percentile were lowest in planned home deliveries. The proportion of weights below the 2.3rd percentile decreased substantially in planned home deliveries after the introduction of the birth centre [2.9 % (95 % CI 1.7–5.0 %) to 0.9 % (0.3–2.2 %)].

After the introduction of the birth centre, standardized intrapartum and early neonatal mortality rate decreased for all the different planned places of birth (taking the period before introduction as standard, see Table 3). Also intrapartum related neonatal morbidity and intrapartum related maternal morbidity rate decreased for all the different planned places of birth. Standardized intervention rates increased for home births and decreased for hospital births, and remained similar for planned birth centre births. Direct and indirect standardization provided similar results.

**Table 3:** Mother and Child outcomes given for planned place of birth (home, hospital and birth centre) after standarizing for parity, age, etnicity and the presence of Small for Gestation Age

Outcome	GCS area		GCS area			Direct standardization								
	Before introduction		After introduction											
	Home	Hospital	Home	Hospital	Birth centre	Home (vs home before intro)			Hospital (vs hospital before intro)			Birth centre (vs hospital before intro)		
	(n)	(n)	(n)	(n)	(n)	95 % CI			95 % CI			95 % CI		
	443	1391	470	1583	1671									
Intra partum and early neonatal death	2	2	0	1	1	0.00	0.00	0.00	0.31	0.00	1.29	0.86	0.00	1.84
Adverse child outcome birth related^a^	13	78	9	43	88	0.62	0.17	1.07	0.46	0.30	0.63	0.81	0.58	1.05
Adverse mother outcomes^b^	37	107	22	118	123	0.52	0.27	0.76	0.96	0.76	1.15	0.90	0.70	1.11
Interventions^c^	48	225	50	215	268	1.32	1.03	1.61	0.89	0.77	1.02	1.01	0.86	1.15

^a^Adverse child outcome birth related is defined as; neonatal encephalopathy, brachius plexus injury, fractured clavicle, cephalohaematoma, neonatal infection low Apgar score (<7 after 5 min), neonatal hospital admission, other trauma related to birth

^b^Adverse mother outcome is defined as; post partum haemorrhage >1000 cc or third or fourth degree rupture

^c^Interventions are defined as; receiving a medical intervention (vacuum extraction, forceps extraction, or caesarean section)

## Comment

The introduction of a midwife-led (homelike) birth centre led to a redistribution of intended place of midwife-led births. Low risk women planning their delivery in the midwife-led birth centre have a higher risk profile compared to low risk women who planned their pregnancy at home or in the hospital, all under supervision of the midwife. This was confirmed by the birth weight distribution. Although the study did not have sufficient power to interpret the observed change in intrapartum and early neonatal death after the introduction of the birth centre (0.22 vs. 0.05 %), the decreasing trend observed in planned home, hospital and birth centre births, suggests on average better care through more adequate selection. A similar trend was observed in intrapartum related neonatal morbidities, decreasing from 5.0 to 3.8 %. Standardized rates showed the largest decrease in intended planned hospital births compared to planned home and birth centre births. A similar trend was observed in intrapartum related maternal morbidities (decreasing from 8.3 to 7.3 %). Standardized rates showed the largest decrease in the planned birth centre births. The rate of interventions in our entire study population was 14.5 %. Intervention rates appeared unaffected by the introduction of the birth centre. Standardized rates of interventions were higher in planned home births, lower in planned hospital births and at the expected rate in birth centre births.

Our cohort study showed some strengths. We used an intention-to-treat-like approach without ex post exclusion of unsuitable midwife cases to create a fair, unbiased comparison of planned home, hospital and birth centre births in this observational context. These unsuitable cases show poorer outcomes, and should therefore be included (van der Kooy et al. 2011).

Our case mix adjustment, using the presence of SGA, proved to be essential. The assumption of comparability across planned places of birth appeared not to be justified, judging from the unequal risk profiles, with home deliveries clearly representing the healthiest group. Self selection by the pregnant women can coincide with implicit or explicit selection by the midwife who may tend to ‘refer’ to a hospital or birth centre if she feels uncomfortable with the risk level at home. Our adjustment of neonatal outcomes with birth weight was done before in similarly standardized analyses (Foster and Kleinman 1982).

Both direct and indirect standardization rates were applied to avoid the effect of accidental outlying subgroups. Direct standardization (weights taken from the index population) gives greater comparability but requires more data. Indirect standardization (weights taken the standard population) requires fewer data but provides less comparability (unless the distribution of the standardization variable is identical across the study populations, in which case standardization is unnecessary since the crude mortality rates could have been compared directly).

As our results show the complete experience of the introduction of a midwife-led birth centre adjacent to the hospital on maternal and perinatal outcome in the north side of Rotterdam, its generelizability is mainly for urban areas. Some study limitations merit discussion. A randomized controlled trail would be the superior design to address our research question. However when homebirth was part of a trial, participation hampered (Tyson 1991; Hendrix et al. 2009) and introduced selective participation which limits generalizability. Moreover if following one’s choice impacts outcome, as expected here, estimates of setting effects are biased too (Tyson 1991; Dowswell et al. 1996). Observational studies as ours are therefore indispensable, despite their shortcomings, in particular the difficulty to overcome the confounding by indication phenomenon.

Secondly, the study did not have sufficient power to assess intrapartum and early neonatal death.

Thirdly, while the presence of SGA was used as additional case mix adjustment, our case mix adjustment could be further improved by detailed risk factors. We cannot rule out remnant confounding by indication as little is known on the factors underlying choice of setting. In addition, we categorized women into Dutch and non-Dutch women for reasons of power, where adverse outcomes may differ among the different ethnic groups [e.g. the increased prevalence of postpartum haemorrhage (Bryant et al. 2012)].

Additionally, no detailed information was obtained on (substandard) care factors, such as decisions for intervention, transferal data (e.g. reasons for transferral to the gynaecologist or traveling time).

Lastly, this study largely neglects the emotional aspects and the aspect of autonomy when comparing places of birth. The choice of the place of birth is largely upon the pregnant woman. The current growth of the share of birth centre and hospital births suggests overall positive balance of these effects, in particular since the economic incentive is in favour of home delivery. Studies assessing the mother’s opinion show that any increase of medical safety easily outweighs other benefits, i.e. emotional aspects (Bijlenga et al. 2009).

Women planning their pregnancy in the midwife-led birth centre apparently have a higher risk profile compared to women who planned their pregnancy at home or in the hospital. A similar trend is observed in the Birthplace cohort (Brocklehurst et al. 2011), while most other international studies show the opposite (Eide et al. 2009; Gottvall et al. 2004; Gottvall et al. 2011; Laws et al. 2010; Waldenstrom and Nilsson 1993). Differential use of these options can be explained by several factors, either intentional or coincidental. After the introduction of the birth centre, low risk women could not plan their delivery in the hospital adjacent the birth centre anymore, but are still able to plan their delivery in other nearby hospitals. This may have led to a shift from the previously planned hospital births to the birth centre. Secondly, our birth centre aims to provide risk led care, with special attention to ethnic minorities and women with a low social economic background. This encourages caregivers to offer the higher risk women (among the low risk group) more explicitly the option of a birth centre delivery. Furthermore, in contrast to planned hospital births, women can receive postpartum care for at least 4 days in the midwife-led birth centre as an option. This may also attract both high risk groups and their caregivers.

The decreasing trend in mortality rate after the introduction of the birth centre (0.22 vs. 0.05 %) should be interpreted with caution due to small numbers. If this, however, truly represents a decrease, it may in part be explained by the beneficial effect of local and national initiatives to lower perinatal mortality, in particular improved risk selection across all delivery options (Denktas et al. 2012a, b; van der Velden 2009). Our study showed a decreasing intrapartum related neonatal morbidity rate in the period after the introduction of the birth centre (5.0 vs. 3.8 %). Standardized rates showed a larger decrease of neonatal morbidity in planned hospital births compared to planned birth centre and home births. This may be due to residual confounding or an actual positive setting effect of planned hospital births.

The total prevalence of intrapartum related maternal morbidities also decreased after the introduction of the birth centre (8.3 to 7.3 %). The decreasing trend observed in planned home, hospital and birth centre births, may suggest on average better care through more adequate selection of women and/or more adequate management, e.g. exact measurement of blood loss and early use of intramuscular oxytocin.

Overall intervention rates were not affected by the introduction of the birth centre. While the underlying pattern suggests better fit of risk profile to setting and a better fit of risk profile to the choice for interventions, the introduction of an extra hospital-based facility did not represent an up- or downward pressure towards intervention rates in general. Previous studies on birth centres showed lower intervention rates combined with an equal, or even better, performance (Gottvall et al. 2004; Gottvall et al. 2011; Laws et al. 2010). These studies, however, did not or only partially adjust for case mix differences (Gottvall et al. 2004; Gottvall et al. 2011; Laws et al. 2010). The few available randomized controlled comparisons also showed lower rates (Hundley et al. 1994; Waldenstrom et al. 1997), or at most an equal intervention rate with equal perinatal outcomes (Eide et al. 2009; Begley et al. 2011; Bernitz et al. 2011). As these trials suffered from non participation or study small size (Hundley et al. 1994; Waldenstrom et al. 1997; Eide et al. 2009; Begley et al. 2011; Bernitz et al. 2011), and showed difficult to combine results (Hodnett et al. 2005), our study adds observational evidence from a large unselected cohort.

## Conclusions

The introduction of a midwife-led birth centre led to a redistribution of women planning their midwife-led delivery at home, at the hospital, or at the local birth centre. Women opting for a delivery in the midwife-led birth centre had the most unfavourable risk profile.

Intrapartum and early neonatal mortality and intrapartum related neonatal and maternal morbidities tended to decrease, while overall intervention rates were unaffected. The introduction of the midwife-led birth centre seems to benefit the outcome of midwife-led deliveries. We interpret this change by the redistribution of the higher risk women among the low risk population intending birth at the birth centre instead of home.
